# In vivo gadolinium nanoparticle quantification with SPECT/CT

**DOI:** 10.1186/s40658-019-0246-y

**Published:** 2019-06-18

**Authors:** Olga Kochebina, Adrien Halty, Jacqueline Taleb, David Kryza, Marc Janier, Alexandre Bani Sadr, Thomas Baudier, Simon Rit, David Sarrut

**Affiliations:** 1 CREATIS-CNRS UMR 5220 - INSERM U1206 - Université Lyon 1 - INSA Lyon - Université Jean Monnet Saint-Etienne, Lyon, 69373 France; 2 Centre Léon Bérard, Lyon, 69008 France; 3 UNIV Lyon - Université Claude Bernard Lyon 1, LAGEPP UMR 5007 CNRS, Villeurbanne, France; 4 Hospices Civils de Lyon, Lyon, 69437 France

**Keywords:** SPECT, Quantification, AGuIX, Nanoparticles, in vivo

## Abstract

**Background:**

Gadolinium nanoparticles (Gd-NP) combined with radiotherapy are investigated for radiation dose enhancement in radiotherapy treatment. Indeed, NPs concentrated in a tumor could enhance its radiosensitization. The noninvasive quantification of the NP concentration is a crucial task for radiotherapy treatment planning and post-treatment monitoring as it will determine the absorbed dose. In this work, we evaluate the achievable accuracy of in vivo SPECT-based Gd-NP organ concentration on rats.

**Methods:**

Gd-NPs were labeled with ^111^In radionuclide. SPECT images have been acquired on phantom and rats, with various Gd-NP injections. Images have been calibrated and corrected for attenuation, scatter, and partial volume effect. Image-based estimations were compared to both inductively coupled plasma mass spectrometer (ICP-MS) for Gd concentration and ex vivo organ activity measured by gamma counter.

**Results:**

The accuracy for the Gd mass measurements in organ was within 10% for activity above 2 MBq or concentrations above ∼ 3–4 MBq/mL. The Gd mass calculation is based on In-Gd coefficient which defines the Gd detection limit. It was found to be in a range from 2 mg/MBq to 2 µg/MBq depending on the proportions of initial injection preparations. Measurement was also impaired by free Gd and ^111^In formed during metabolic processes.

**Conclusions:**

Even if SPECT image quantification remains challenging mostly due to partial volume effect, this study shows that it has potential for the Gd mass measurements in organ. The main limitation of the method is its indirectness, and a special care should be taken if the organ of interest could be influenced by different clearance rate of free Gd and ^111^In formed by metabolic processes. We also discuss the practical aspects, potential, and limitations of Gd-NP in vivo image quantification with a SPECT.

## Introduction

The use of nanoparticles (NP) in radiotherapy may help to enhance the dose ratio between a tumor and healthy tissues [1]. The nanoparticles based on high-Z metals are delivered to a tumor and generate a localized absorbed dose enhancement within the target volume. Indeed, it has been shown on simulations that when the X-ray beam hits densely packed gold NPs, the photoelectric effect increases, leading to the emission of additional electrons depositing their energy locally [2]. It has been proposed to use Gd nanoparticles for tumor radiosensitization [3, 4]. Many research and industrial groups are actively working on this subject [1, 5, 6]. However, despite good in vitro results, in vivo performances are still controversial and actively investigated. Two of the main concerns are the in vivo localization and the quantification of the NP. These are crucial tasks to determine the delivered dose for radiotherapy treatment as radiosensitization would change the optimal dose to deliver.

In the past two decades, the main effort was either on only qualitative in vivo imaging or in vitro/ex vivo quantification of NPs. For example, inductively coupled plasma mass spectrometer (ICP-MS) techniques [7] can provide quantitative elemental composition with sensitivity of 1 ng/L [8]. The ICP-MS detects ions distinguished by their mass-to-charge ratio in incinerated samples, and therefore, it provides destructive tests and cannot be used in vivo. Optical imaging [9] offers high sensitivity and uses non-ionizing radiation but low penetration depth. Moreover, this method is non-quantitative for in vivo imaging studies [9].

Magnetic resonance imaging (MRI) seems to be a good candidate for localization and quantification of metal NPs, particularly gadolinium NPs (Gd-NPs) [10]. It has a high image resolution and excellent soft tissue contrast. The Gd contrast agents are widely used to improve MRI images. However, due to nonlinear correlation between the contrast agent concentration in tissue and the MRI signal, in vivo quantification remains challenging. A recent study [10] shows that the protocol for quantitative MRI should take into account the age of the patients and their diagnoses. It is also prudent to keep the MR field strength constant and the same T1 weighted sequence for all patients. Moreover, one of the major limitation of MRI is a lack of sensitivity for low concentration of Gd [10].

The radionuclide-based imaging such as positron emission tomography (PET) combined with MRI was also tested for image-guided radiation therapy [11]. The Gd-NPs can be radiolabeled with ^68^Ga (*T*_1/2_=68 min) tracer and, thus, be detected and quantified from PET images. However, the authors of [11] only provided qualitative assessment for PET images without quantitative image analysis. Another nuclear medicine technique that could be used is single photon emission computed tomography (SPECT) where the Gd-NPs could be labeled with ^111^In (*T*_1/2_=2.8 days) tracer. Even if PET quantification might be easier to implement, SPECT image quantification [12–14] is also possible. Widely available in clinic, *β*+ emitters (^18^F, ^68^Ga) have relatively short half-life (*T*_1/2_=110 min and *T*_1/2_=68 min) compared to ^111^In. Other *β*+ emitters such as ^64^Cu or ^89^Zr have longer half-life but are not always available. Here, we focus on SPECT with ^111^In, which was the only available modality at our disposal.

In this work, we aim to quantify the in vivo Gd concentration distribution of AGuIX Gd-NPs [6] labeled with ^111^In from SPECT images. Image-based estimations were performed on phantoms, rats kidneys, and chondrosarcomas tumors. Obtained values were compared to ICP-MS measurements. The main steps are described in the “Method and materials” section, followed by results and discussion as well as difficulties that could be avoided in future similar researches.

## Method and materials

### General workflow

**Fig. 1:**
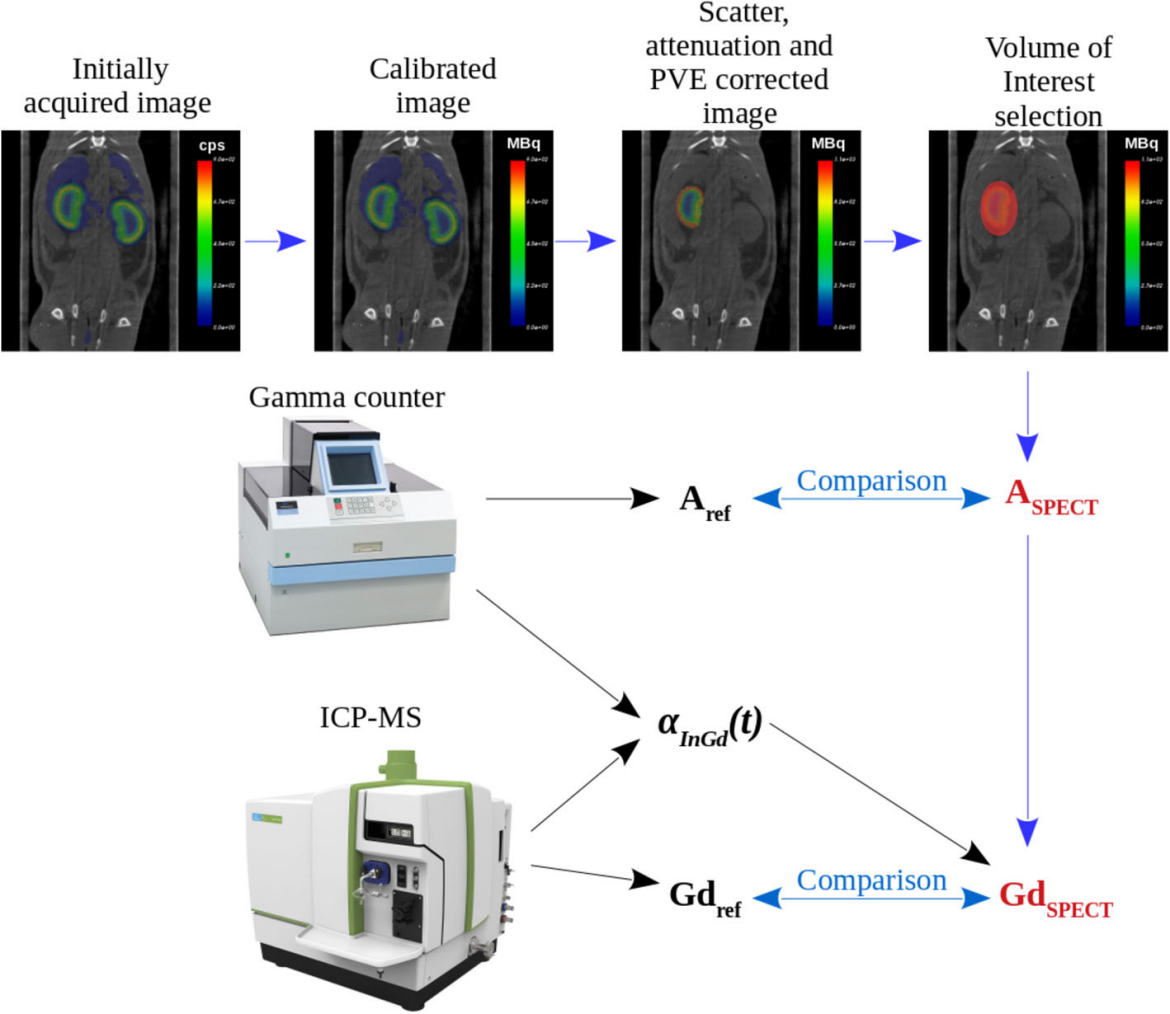
Main steps of the analysis. Schematic representation of the main steps of the analysis

The quantification of Gd mass in organ from SPECT images is an indirect process, see Fig. 1. First, the ^111^In concentration in a volume of interest (in red in Fig. 1) was estimated from images that were calibrated and corrected by scatter, attenuation, and partial volume effect. An ^111^In to Gd factor *α*_*InGd*_ was estimated and applied to derive the corresponding Gd mass. Obtained values were compared for activity to reference gamma counter and for Gd mass to ICP-MS measurements. Studies were performed on both phantom and rat images acquired on a preclinical SPECT/CT device.

### Nanoparticle radiolabeling

The AGuIX nanoparticles used in this study were obtained from NHTheraguix (Crolles, France). They are composed of a polysiloxane matrix bearing DOTA chelators on the surface able to chelate Gd3+ ions and ^111^In for SPECT experiments. The AGuIX NP hydrodynamic diameter is under 6 nm. The nanoparticles (50µL, 100 mM) were radiolabeled by adding 300 µL of citrate buffer 50 mM pH5 and 40–80 MBq of high purity ^111^In-chloride (Mallinckrodt, Petten, Netherlands). The mixture was incubated for 30 min at 40 ^∘^C. A diethylenetriaminepentaacetic acid (DTPA) was added at the end of radiolabeling after incubation for free ^111^In evacuation. Radiochemical purity of AGuIX-^111^In was over 97%.

For stability testing, an aliquot of the radiolabelled AGuIX-^111^In was incubated at 37 ^∘^C in 2 mL phosphate buffer saline (pH 7.4) and in rat serum, and radiochemical purity (RCP) was evaluated using ITLC-SG and citrate buffer 0.1M pH5 as mobile phase. This test showed that at 48h after incubation, RCP was still greater than 96% in phosphate buffer saline (pH 7.4) and in rat serum indicating a suitable kinetic stability to perform in vitro and in vivo experiments.

### Phantoms and animals

For the in vitro studies, two sets of data were analyzed:

- ^111^In + Gd-NPs. Four tubes were used, containing 500 µL of saline solution with different concentrations of Gd-NPs labeled with ^111^In corresponding to 7.46 MBq, 4.20 MBq, 2.16 MBq, and 1.23 MBq at the SPECT imaging time. They were used to measure the Gd mass (described below).
- ^111^In without Gd-NPs. Six tubes were imaged, containing 250 µL of ^111^In corresponding to 9.85 MBq, 4.55 MBq, 2.50 MBq, 1.27 MBq, 0.67 MBq, and 0.37 MBq at the SPECT imaging time. The aim of this experiment was to evaluate the linearity of the image-based quantification for different activities.

For in vivo imaging, 9 OFA (Oncins France Strain A) male rats with chondrosarcomas were used, four weeks after tumor placement. The animals were injected both intratumorally or intravenously with an ^111^In radiolabeled Gd-NPs in the presence of DTPA, with activities ranging from 5 to 25 MBq measured for each injection with a dose calibrator (Capintec Inc., Florham Park, USA). Animals were sacrificed at 5 min and 30 min intratumorally and 1, 2, and 4 h for intravenously injected animal. The original study plan included measurements at 6H and at 24H. Even if the injected activity was not low (10–20 MBq), only the image obtained at 4H were usable while the signal-to-noise ratio of the others was insufficient. We also stop at 4H point because the full irradiation study, that is under publication, showed that the treatment is effective at this time point. Images were acquired post mortem in order to have reference measurements on extracted organs. Kidneys and tumors were removed and placed in formol in plastic tubes adapted for a gamma counter measurement of the activity. Supplementary images were also acquired on these tubes for ex vivo studies.

### SPECT/CT image acquisition

We used a nanoSPECT/CT (Bioscan Inc., Washington D.C., USA) for preclinical imaging with multiplexing multipinhole apertures. It has four detection heads allowing the acquisition of four projections simultaneously. The pinhole collimator for rat imaging used in the experiments, named APT2, has 9 cone shape pinholes drilled from both sides of the collimator giving an opening diameter of 2.5 mm. The field of view of the camera is a cylinder with a diameter of 65 mm, and the axial scan length of 25 mm. We used ^111^In radionuclide emitting 171.3 keV (90.61%) and 245.4 keV (94.12%) gamma rays. Therefore, the projections were acquired for two energy windows of 10% around the peaks and one additional energy window of 209 keV ± 10% used for scatter correction.

The SPECT device acquired 24 projections (6 projections × 4 heads) of 256 ×256 pixels for every 15 degrees. The scan duration was 100 seconds per projection. The reconstruction was performed with the manufacturer software, HiSPECT, using an ordered subsets expectation maximization (OSEM) algorithm with 9 iterations and 4 subsets with an image voxel size of 0.6 mm.

The cone-beam CT scans contained 180 projections for the full coverage with a duration of 1 s/projection acquired with a beam voltage of 55 kV. The images were reconstructed with Feldkamp’s filtered backprojection reconstruction algorithm [15] with a voxel size of 0.4 mm. The reconstructed CT images were registered and resampled in order to match the sampling of the SPECT images.

### SPECT image corrections

#### Scatter

The dual-energy correction method (DEW) [16] was used for the scatter corrections. This method consists in subtracting an estimate of the scatter component from the peaks. It is based on a measurement of the number of counts in one energy region near the peak. The two peaks, 171.3 keV and 245.5 keV, were corrected for the scatter component based on the number of counts in the scatter window between these peaks.

#### Attenuation

The reconstructed SPECT images were corrected with Chang’s multiplicative method [17] on a voxel-by-voxel basis. The linear attenuation coefficient images were recalculated from CT images using a bilinear model (see [18]) and based on the NIST tables of mass attenuation coefficients. The attenuation correction factors (ACF) for each voxel of the reconstructed image were obtained taking into account the gamma path through the tissue.

The ^111^In isotope has two photopeaks which means that the attenuation correction should be done for these two peaks separately. However, the comparison between the correction for two peaks simultaneously and separately gave a difference of ∼ 1%. Therefore, we applied the attenuation corrections in the two energy windows by calculating a weighted combination of the ACFs with experimentally defined weights for each peak component as *w*_171keV_=0.635 and *w*_245keV_=0.365.

#### Partial volume effect

SPECT images suffer from PVEs due to limited spatial sampling and a finite spatial resolution [19]. Therefore, a region of high activity tends to be underestimated and neighboring voxels overestimated. This means that if the VOI is selected from an anatomic CT image, the measured activity will be biased (underestimated in this case). In this study, we used post-reconstruction Müller-Gärtner method (MGM) [20] for partial volume effect correction in its generalization to two regions (see [21] for the detailed workflow).

### Absolute calibration

For NanoSPECT/CT image calibration, we followed the NEMA standard protocol [22]. We used a cylindrical phantom with 5-mL volume containing the activity with a concentration of *c*_Vol_=0.72±0.01 kBq/mL measured with a gamma counter (Wallac Wizard 1470 Gamma Counter, GMI inc.) and recalculated for the acquisition time. The calibration system volume sensitivity, *S*_Vol_ [23] (in cps/Bq), was calculated as:

$${} S_{\text{Vol}}\,=\,\frac{R}{c_{\text{Vol}}\cdot V_{\text{VOI}}}\times \text{exp} \left(\frac{T_{0}-T_{\text{cal}}}{T_{1/2}}\cdot \mathrm{ln2}\right)\\\!\times\!\left(\frac{T_{\text{acq}}}{T_{1/2}}\cdot \mathrm{ln2}\right)\times \left(1-\text{exp}\left(-\frac{T_{\text{acq}}}{T_{1/2}}\cdot \mathrm{ln2}\right)\right)^{-1},$$

where *V*_VOI_ (in mL) is a volume of interest (VOI) placed in the reconstructed image, *T*_0_ is the start time, *T*_acq_ is the duration of the acquisition, *T*_1/2_ is the half-time of the radionuclide used, *T*_cal_ is the time of the activity calibration, and *R* (in cps) represents the counting rate measured in the VOI.

### Volume of interest selection

For phantom and ex vivo studies, we used CT images for VOI selection. The difference of HU values for water and soft tissue and air provided an opportunity to obtain VOI by binarization of CT images with an adapted threshold.

**Fig. 2:**
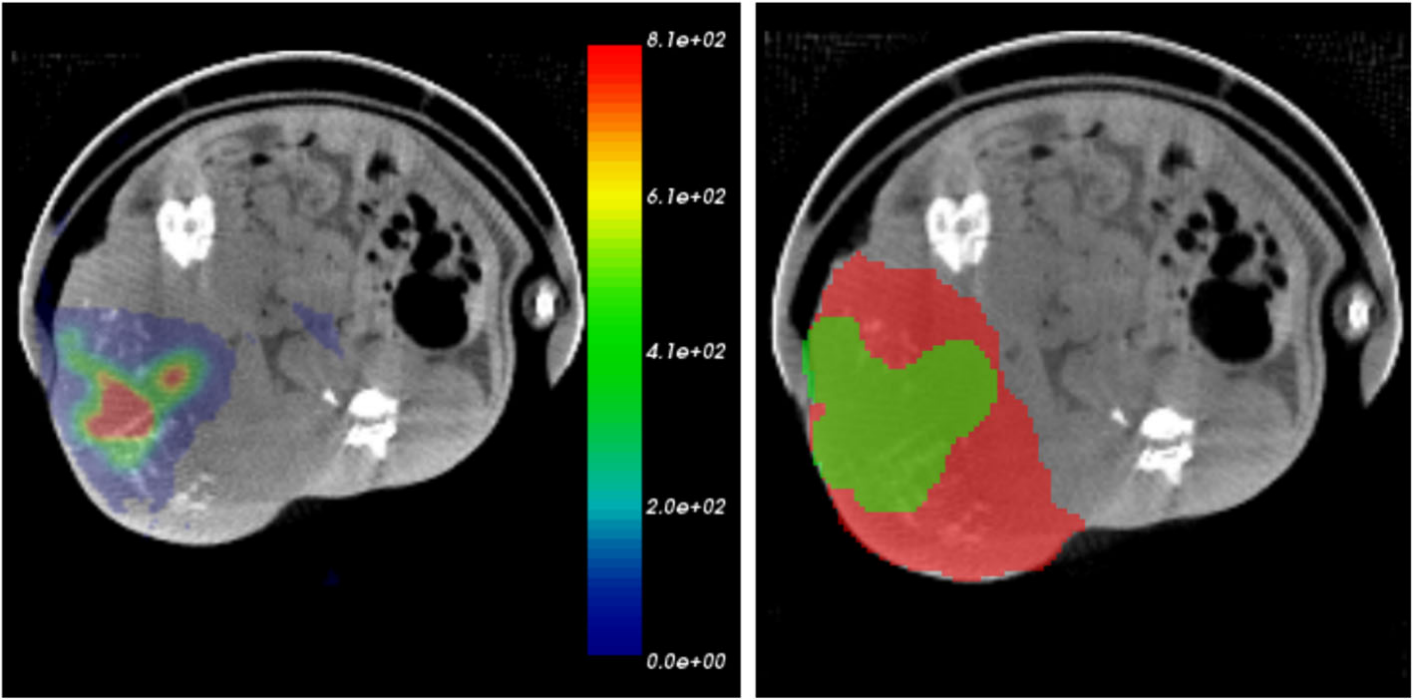
Possible choice of VOI selections. Illustration of in vivo SPECT/CT tumor image with ^111^In activity (left) and possible choice of VOI selections (right): total volume of tumor (red) and VOI from SPECT image containing only radioactivity perfusion region (green)

For in vivo studies, the separation of kidneys and tumors from surrounding soft tissues was difficult in CT images. Therefore, the SPECT images were used for the VOI selection. For kidney studies, we first applied a threshold in SPECT images, and then, as a spill-out from the PVE would bias this selection, we eroded the VOIs in order to match the borders in CT images. For the tumor analysis, the threshold in SPECT images cannot be used in the same manner as the activity distribution was heterogeneous and its border mismatched the actual tumor borders in anatomical image (Fig. 2). The whole tumor VOI is presented in Fig. 2 in red and the VOI selected for activity above certain threshold is shown in green. Such VOIs were selected in SPECT images following these steps: (i) image with Gaussian filtering with *σ* of 1.5×(*voxel* *size*), (ii) define the threshold as 8% of a maximum value in a local region in the blurred image, and (iii) dilate the obtained VOI by 2 voxels. This approach has been approved in kidney images before using it in the final tumor image analysis.

### Gadolinium quantity calculation

The obtained ^111^In activity measurement from SPECT images was used to find the equivalent Gd-NP quantity. The coefficient of proportionality between these two values, *α*_*InGd*_(*t*), was defined as

$$\alpha_{InGd}(t)=\frac{m_{Gd}}{A_{In}(t)},$$

where *m*_*Gd*_ is the mass of Gd-NP and *A*_*In*_(*t*) is the ^111^In activity at time *t*. It could either be measured or calculated for each injection preparation. In these studies in case of measured *α*_*InGd*_(*t*), the activity, *A*_*In*_(*t*), was obtained with the Wallac Wizard gamma counter and the Gd mass, *m*_*Gd*_, was determined by ICP-MS. We also used calculated $\alpha ^{calc}_{InGd}(t)$ coming from the same formula as above from initial proportions of gadolinium mass, *m*_*Gd*_, and ^111^In activity at preparation time.

In the experiments on phantom tubes, *α*_*InGd*_(*t*) was obtained on the sample with the highest concentration and used to calculate the Gd-NP concentration for the three other tubes. In in vivo kidney studies, several independent samples from the same preparation as in the main analysis were used to determine *α*_*InGd*_(*t*) by fitting the linear proportionality between *A*_*In*_(*t*) and *m*_*Gd*_. In in vivo tumor studies, we also used the calculated $\alpha ^{calc}_{InGd}(t)$.

### Reference measurements for ^111^In and Gd-NPs

In order to evaluate the SPECT image-based activity quantification, we measured the reference values on the same Wallac Wizard gamma counter as for the calibration. The reference values of the Gd masses were obtained with the ICP-MC measurements. This study was originally made on post mortem animals because the reference values of the activity could only be measured on extracted organs.

### Uncertainties estimation

In order to estimate the uncertainty on the activity measurements we summed up in quadrature the following individual uncertainties:

- Standard deviation of a count rate in VOI
- Uncertainty on mask selection was taken of 10%
- Uncertainty on activity reference measurement with a gamma counter (2%)

## Results

### Calibration for ^111^In quantification

The calibration system volume sensitivity after scatter, attenuation, and PVE corrections mentioned above was *S*_Vol_=(2.11±0.04)·10^3^ cps/MBq. This calibration coefficient was used for in vitro, in vivo, and ex vivo image quantification.

### Phantom studies

**Fig. 3:**
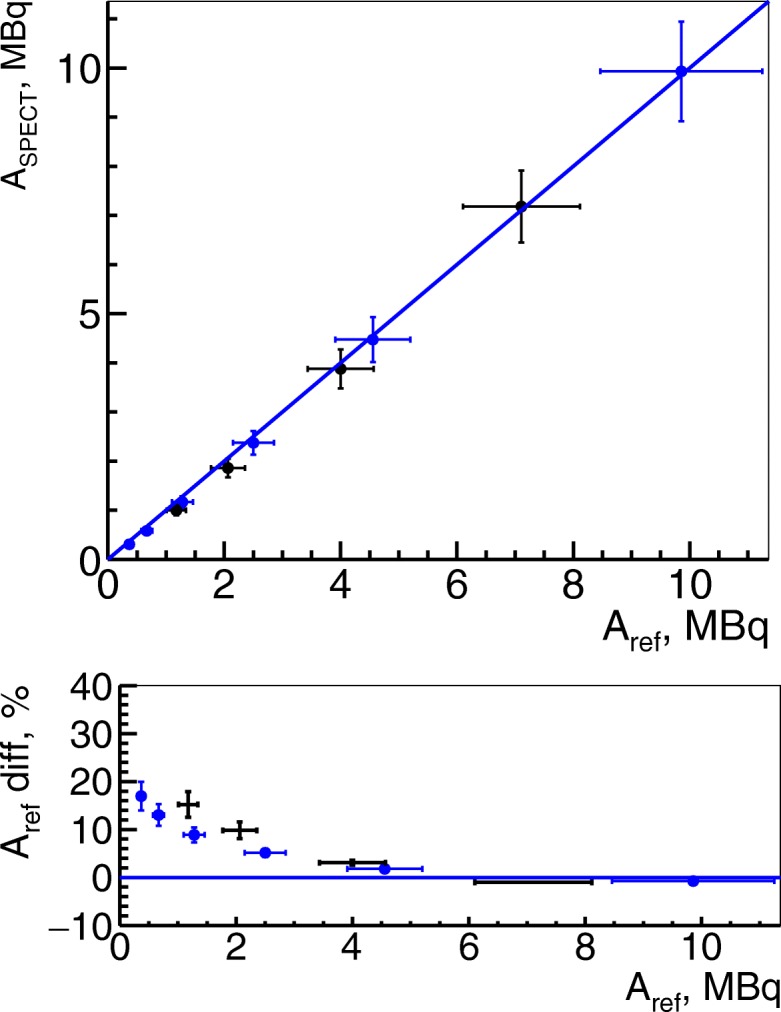
Activity from SPECT images in phantom studies. Activity measured from corrected SPECT images in phantom studies for ^111^In + Gd-NPs (black points) and ^111^In without Gd-NPs (blue points). The error bars for measured activities are the standard deviations

Results on image quantification with system volume sensitivity given above is presented in Fig. 3, where one can observe two sets of points: black for four ^111^In + Gd-NPs samples and blue for six ^111^In without Gd-NPs as explained above. A linear correspondence between measured and reference activities was established for two independent sets of measurements (Fig. 3, top). The accuracy shown at the bottom of Fig. 3 is larger than 10% for activities below 2 MBq. In terms of concentration, it corresponds to 4–8 MBq/mL as the volumes of images tubes were 250µL and 500 µL.

**Fig. 4:**
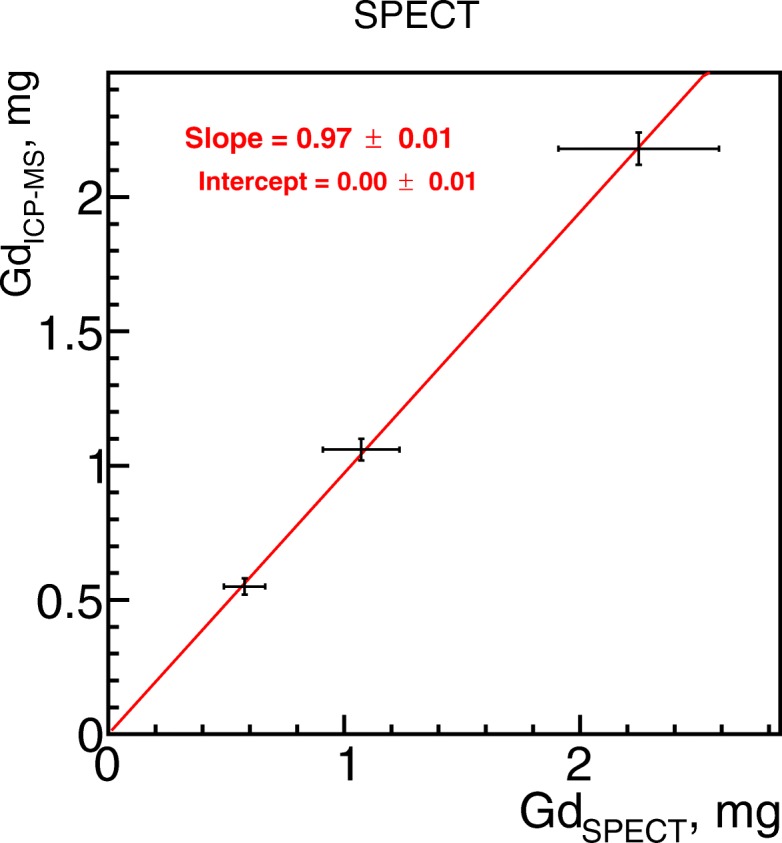
Gadolinium mass from SPECT images in phantom studies. Gadolinium mass measurement from SPECT images for ^111^In + Gd phantom samples as a function of expected mass of gadolinium measured with ICP-MS

The calibration coefficient for Gd masses *α*_*InGd*_(*t*_*img*_) was (0.56±0.06) mg/MBq. The measurements of Gd mass on the ^111^In + Gd samples are presented in Fig. 4 and show linear dependence between measured and reference values.

### In vivo studies on kidneys

**Fig. 5:**
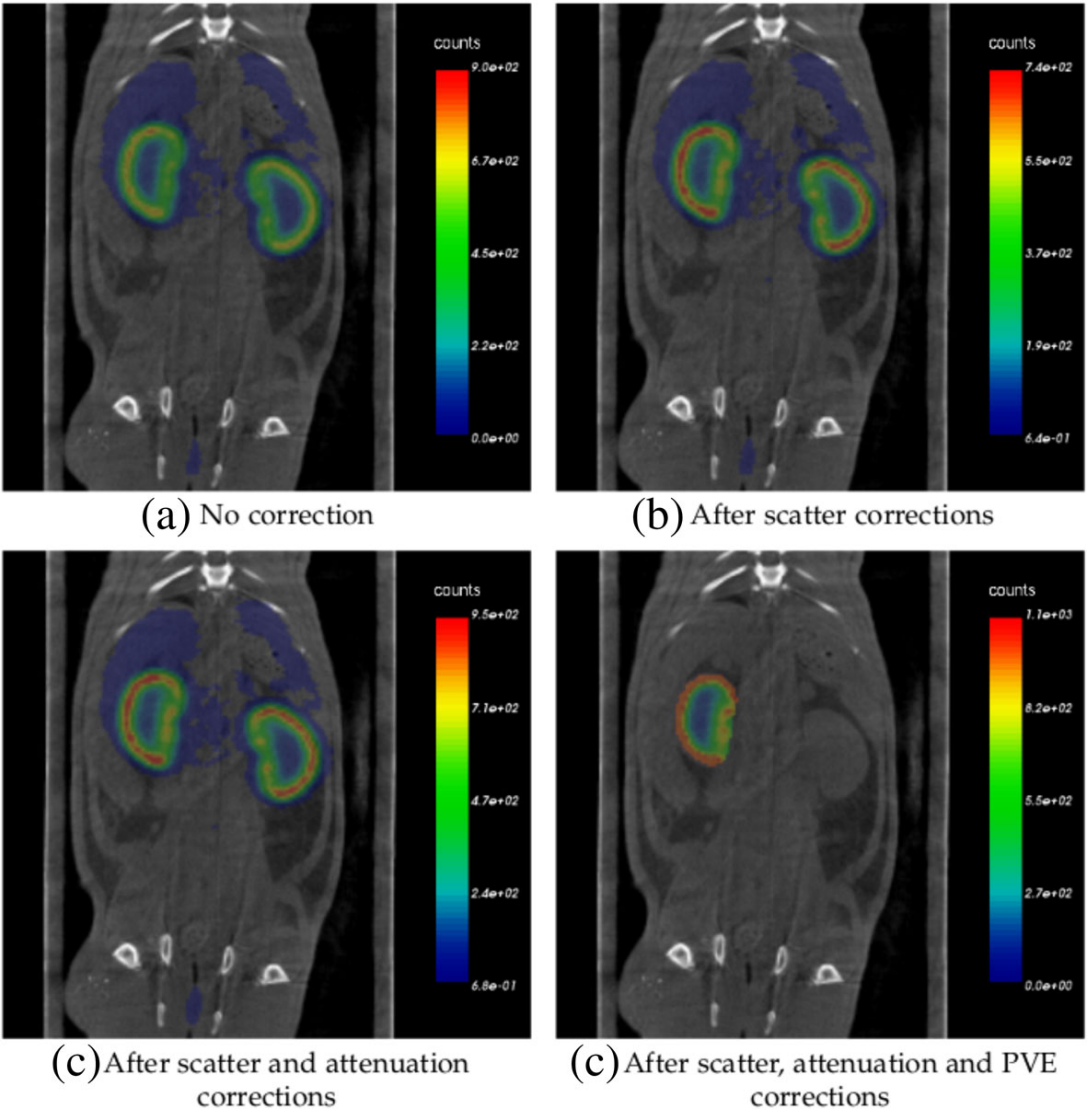
Examples of in vivo images. Examples of in vivo images with no corrections(**a**), after only scatter corrections(**b**), after scatter and attenuation corrections(**c**), and after scatter, attenuation and PVE corrections (**d**). The activity outside the target VOI has been masked out during PVE corrections

The illustration of the result of SPECT image corrections is presented in Fig. 5, where one can find SPECT/CT images with no correction, after only scatter corrections, after scatter and attenuation corrections, and after scatter, attenuation, and PVE corrections. The last one masks out the activity outside of target VOI.

**Fig. 6:**
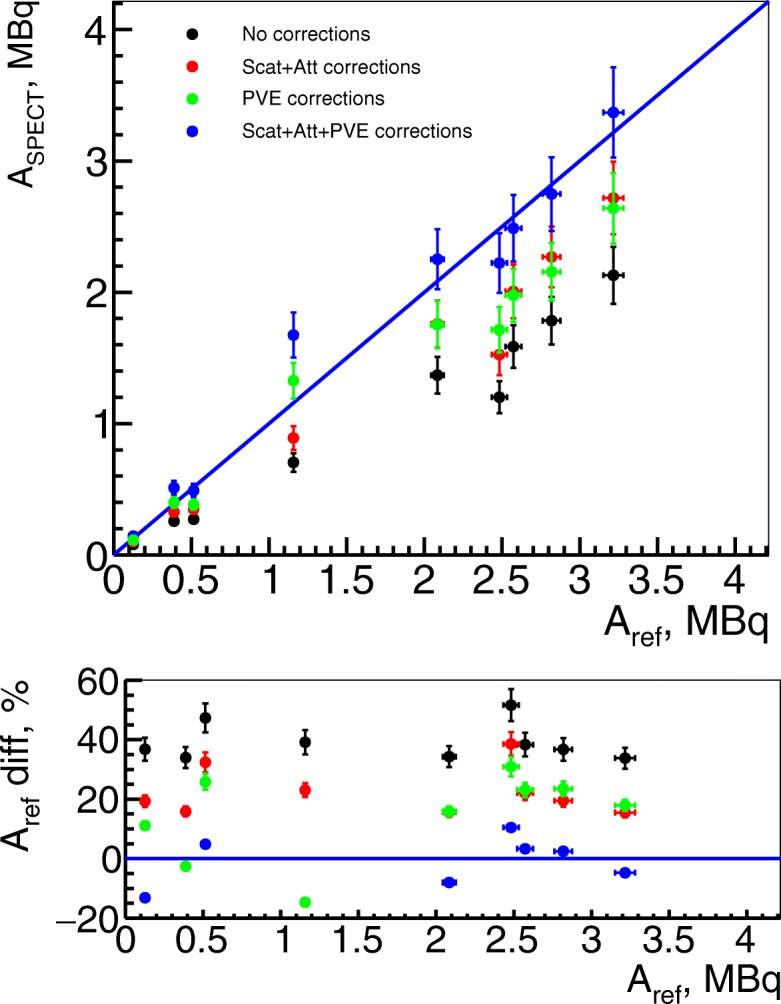
Activity from SPECT images in in vivo studies (kidneys). In vivo ^111^In activity measurements on right kidneys from uncorrected images in black, from images with only attenuation correction in red, with only PVE corrections in green and with all corrections in blue

**Fig. 7:**
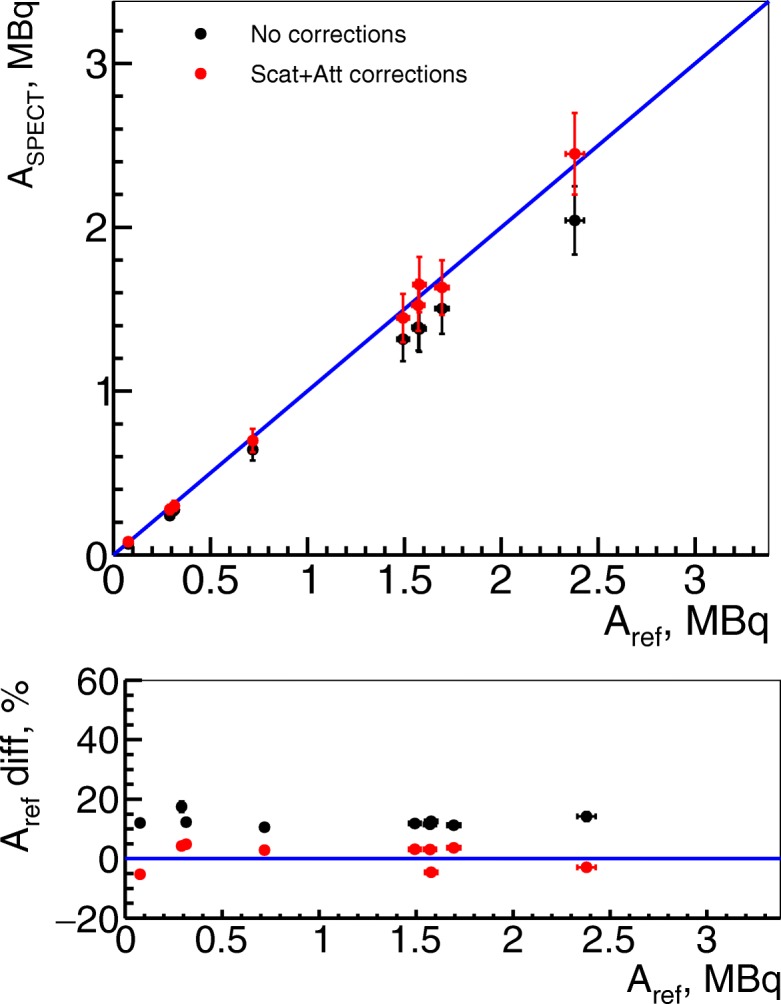
Activity from SPECT images in ex vivo studies (kidneys). Ex vivo measurements on right kidneys of ^111^In activity for uncorrected images in black and with only scatter and attenuation correction in red. The PVE corrections were not applied as volume of interest containing a kidney, and a formol was large enough to take into account this effect

The results of activity quantification on right kidneys are presented in Fig. 6 for in vivo and in Fig. 7 for ex vivo studies. In these plots, one can observe that after attenuation, scatter, and PVE corrections, in vivo quantified activities correspond to their expected values. However, the total activity in kidneys was below 3.5 MBq while, from the in vitro tests (Fig. 3), the quantification becomes non-linear below 2 MBq. The in vivo results also differ from reference within 10% for the activities above 2 MBq, which confirms the results on tube phantoms.

**Fig. 8:**
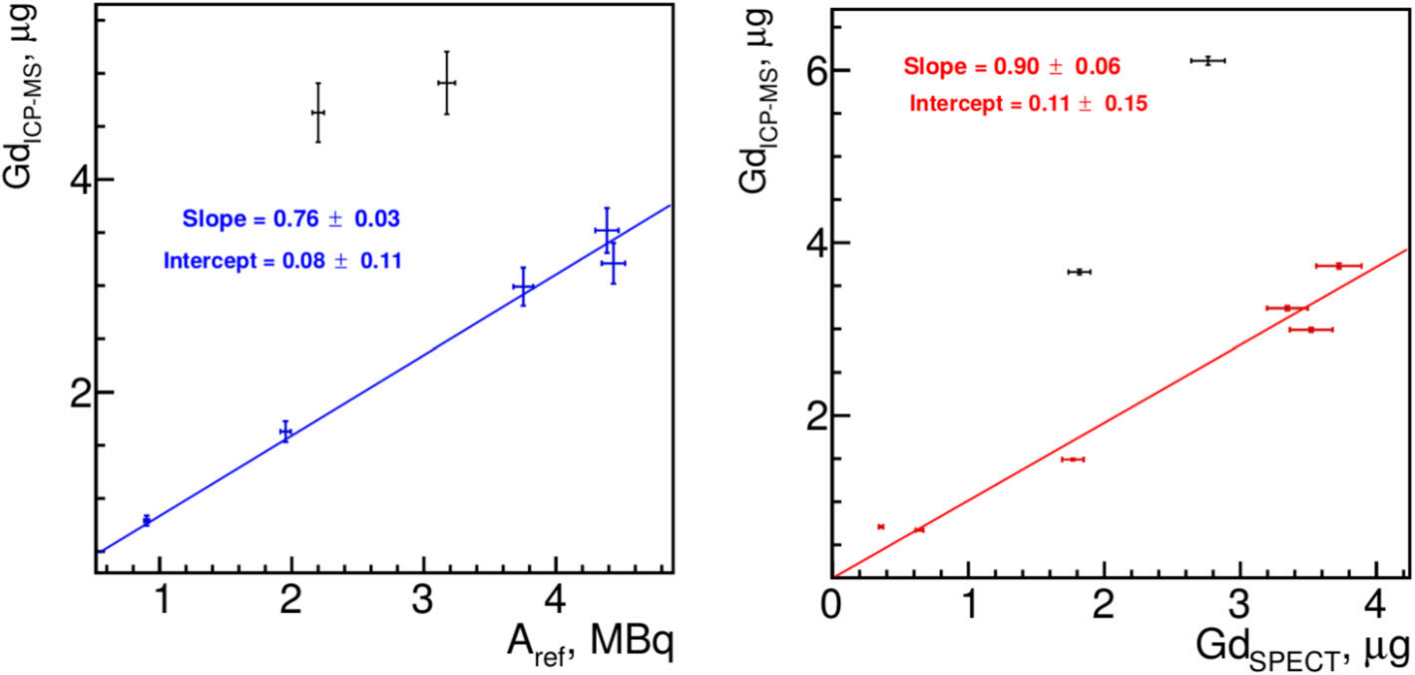
Calibration and result for gadolinium mass measurement from SPECT images for in vivo studies (kidneys) Left: The *α*_*InGd*_(*t*_0_) coefficient at injection preparation time for in vivo studies based on left kidneys. Right: Gadolinium mass measurement from SPECT images for in vivo studies vs. expected mass of gadolinium measured with ICP-MS in right kidneys. The errors on slope and intercept are only fit errors without taking into account the systematic uncertainties from SPECT image calibration, *A*_*ref*_ and G*d*_*ICP*−*MS*_ measurements

The result of *α*_*InGd*_(*t*_0_) obtained on left kidneys at injection preparation time is presented in Fig. 8, left. It could be observed that two points are out the general behavior which is discussed in the “Discussion” section below. We decided to exclude two points from the linearly fit which were obvious outliers for both kidneys.

The measured *α*_*InGd*_(*t*_0_)=(0.76±0.03) µg/MBq at preparation time was recalculated for in vivo imaging moment. The result of in vivo Gd mass quantification is presented in Fig. 8, right where Gd quantity obtained from SPECT images were compared to ICP-MS reference measurements. Once again, the two kidneys from the same animals are out of the linear fit.

### In vivo studies on tumors

**Fig. 9:**
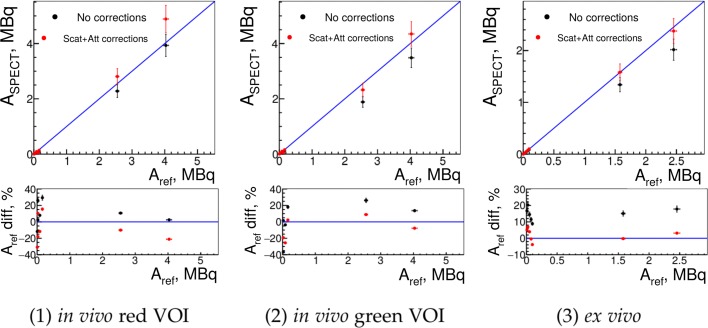
Activity from SPECT images in in vivo and in ex vivo studies (tumors). The image-based activity measurements on tumors for uncorrected images (black) and after attenuation correction (red). From left to right: (1) on in vivo in the red VOI of Fig. 2; (2) on in vivo in the green VOI of Figure 2; (3) on ex vivo

The results of image-based activity quantification in red-type VOIs and green-type VOIs from Fig. 2 are presented in Fig. 9, left and middle, correspondingly. The total activity measured in green-type VOIs for the two highest concentrations is within 10% of the expected reference value. Ex vivo results on tumors show the accuracy within 5% for the activities above 1 MBq (Fig. 9, right).

For the Gd mass quantification, we had the reference value only for one point in Figs. 9 at 2.55 MBq. With *α*_*InGd*_(*t*_0_)=(0.76±0.03) µg/MBq obtained on left kidneys (Fig. 8, left), the image-based measurement gave (3.1±0.2) µg while the ICP-MS reference was (4.58±0.05)µg, which is the same order of magnitude but differs by almost 70%. However, the $\alpha ^{calc}_{InGd}(t_{0})$ calculated from the initial proportions of Gd-NPs and ^111^In was (0.91±0.03) µg/MBq and gave (4.1±0.2) µg which differs only by 10% from the reference value.

## Discussion

In this article, we presented a method for Gd nanoparticle quantification from SPECT images when they are labeled with ^111^In. Once all corrections (scatter, attenuation, PVE) are applied, we showed that image-based SPECT quantification of total Gd mass in organs is feasible and could be 10% accurate for the total activities above 2 MBq, i.e., 4 MBq/mL in terms of concentration. The studies on phantoms supported the hypothesis that SPECT images could be used for Gd-NP quantification as a linear correspondence between measured from SPECT images and ICP-MS reference masses of Gd was observed. We obtained ∼ 2 mg/mL of Gd-NP concentration as a lower limit for 10% accuracy measurements.

The in vivo image quantification of Gd-NPs after scatter, attenuation, and PVE corrections also confirms that hypothesis. Considering the volume of the kidney cortex (the region where the tracer was mainly accumulated) about 0.7 mL, we obtained the activity concentration of the same order of magnitude as for phantom experiment: ∼ 3 MBq/mL. At the injection preparation time, *α*_*InGd*_(*t*_0_) was 0.76±0.03 µg/MBq which gives the detection limit of Gd at the concentration of ∼2 µg/mL which is 3 order of magnitude lower than in phantom experiment. This shows that by adjusting the In-Gd proportions, one can choose the detectable concentration of Gd-NPs at least in a range from 1 mg/mL to 1 µg/mL while keeping the 10% accuracy defined by ^111^In image-based measurement.

We observed in in vivo studies that two kidney points were out of the linear fit behavior. It is hypothesized that due to use of DTPA, the different kidneys’ clearance rate of free Gd and ^111^In formed during metabolic processes could explain this observation (more details, for example, in [24]). The same two animals showed this behavior for *α*_*InGd*_(*t*) measurement in their left kidneys and also for Gd masses obtained in their right kidneys which is in line with the hypothesis of different clearance rate for different animals. This observation revokes the developed SPECT quantification approach as the ^111^In activity measured in images is in line with reference values. However, due to indirectness of the Gd-NP mass quantification, metabolic processes can influence the *α*_*InGd*_(*t*) and, thus, the eventual measurement. This means that even if kidneys provide most of the time the largest image signal, the tests of quantification approaches for Gd quantification should be done on other organs, preferably on tumors.

The in vivo image quantification on tumors showed the crucial influence of the VOI choice. We tested methods to define them from anatomical or functional images. We can conclude that the strategy should be adapted to concrete activity distribution properties. Also, the in vivo image quantification for Gd-NPs on tumors demonstrated that the obtained values based on *α*_*InGd*_ measured on kidneys were less consistent with expectation than the result based on $\alpha ^{calc}_{InGd}$ calculated from initial proportions. This is again in line with a hypothesis about different clearance rate of free Gd and ^111^In.

All this demonstrates the main limitation of the method. As in vivo Gd measurements are indirect as one can measure only ^111^In activities from SPECT images and deduce Gd masses from them. The coefficient between these two values, *α*_*InGd*_(*t*), should be precisely known and that the labeling of ^111^In and Gd should be stable in time and evacuated simultaneously from an organ. Otherwise, the Gd mass measurement from SPECT images could be unreliable.

An alternative to SPECT imaging for the same aim could be PET as AGuIX nanoparticles can be labeled with ^68^Ga PET tracer. This modality could potentially be more precise for image quantification but remains also indirect but probaly is less affected by methabolism processes as chemical labeling is slightly different. For direct Gd concentration measurements from images, a new modality, Spectral Photon Counting CT (SPCCT), could be used [25, 26]. SPCCT is a X-ray tomographic acquisition system using dedicated detectors in photon-counting mode with energy discrimination. Energy thresholds could be set such that optimal discrimination of Gd is obtained.

During this work, we faced several problems that could be avoided in the future. We propose several recommendations for protocol developments and tests for in vivo of Gd quantification methods:

- The chemical bounding of Gd nanoparticles with a radionuclide has to be done without purification, as this is important for the calculation of the In-Gd coefficient *α*_*InGd*_(*t*), which can vary from mg/MBq to µg/MBq.
- Tests of Gd quantification methods of nuclear medicine images done on kidneys are unreliable as the In-Gd recalculation coefficient *α*_*InGd*_(*t*) could be modified by metabolic effects.
- Special care should be taken for VOI definitions. We advise to do a threshold on anatomical or functional images depending on difference of HU values inside and outside of VOI in CT images and on the heterogeneity of activity distributions. The detailed protocol for the VOI definition is proposed in the “Method and materials” section.
- The use of formalin should be avoided on ex vivo samples. It makes the testing of VOI selection difficult as the HU values of formalin is similar to the one of soft tissues. Additionally, the tests of partial volume effect corrections become impossible. Moreover, the use of formalin complicates ICP-MS analysis and makes it less accurate. An alternative for ex vivo sample conservation could be freezing.

## Conclusion

In this paper, we studied the potential of SPECT imaging for in vivo quantification of gadolinium nanoparticles. The main motivation was the possible application of this method for the treatment planning of radiotherapy enhanced by Gd-NPs. Even if SPECT image quantification remains technically difficult, it has potential for precise Gd concentration measurements. It is indirect, and thus, its accuracy is mainly defined by ^111^In image quantification accuracy and the coefficient of proportionality between Gd and ^111^In. We observed that the accuracy of the ^111^In image quantification is better than 10% for activity above 2 MBq or concentrations above ∼ 3–4 MBq/mL. The In-Gd coefficient to calculate Gd from ^111^In images may vary at least from mg/MBq to µg/MBq which defines the Gd-NPs detection limit from mg/mL to µg/mL (from ∼2 mg/mL to ∼2 µg/mL in this study) with accuracy of 10%.

We showed that the SPECT image quantification method is accurate. However, the main limitation comes from disproportionality of *α*_*InGd*_ in time in different organs. We assume that the clearance rate of free Gd and ^111^In formed by metabolic processes could be different. Therefore, the results obtained in organs in question, such as kidneys, could be unreliable. Yet, this does not defeat the image-based Gd-NP measurements in tumors which seems to be unaffected by this problem.
